# Detecting central sleep apnea in adult patients using WatchPAT—a multicenter validation study

**DOI:** 10.1007/s11325-019-01904-5

**Published:** 2019-08-11

**Authors:** Giora Pillar, Murray Berall, Richard Berry, Tamar Etzioni, Noam Shrater, Dennis Hwang, Marai Ibrahim, Efrat Litman, Prasanth Manthena, Nira Koren-Morag, Anil Rama, Robert P. Schnall, Koby Sheffy, Rebecca Spiegel, Riva Tauman, Thomas Penzel

**Affiliations:** 1 Technion Faculty of Medicine, Sleep Laboratory, Carmel Medical Center, Haifa, Israel; 2 Center of Sleep and Chronobiology, Toronto, ON Canada; 3 Health Sleep center, University of Florida, Gainesville, FL USA; 4 Cardiology Department, Soroka Medical Center, Be’er Sheva, Israel; 5 Kaiser Permanente Fontana Medical Center, Fontana, CA USA; 6 Cardiology Department, Rambam Medical Center, Haifa, Israel; 7 Itamar Medical, Caesarea, Israel; 8 Sleep clinic, Kaiser Permanente Los Angeles Medical Center, Los Angeles, CA USA; 9 Sackler School of Medicine, Tel Aviv University, Tel Aviv-Yafo, Israel; 10 Kaiser Permanente San Jose Medical Center, San Jose, CA USA; 11 Stony Brook University Hospital, Stony Brook, NY USA; 12 Sleep Disorders Center, Tel Aviv Souraski Medical Center, Sackler School of Medicine, Tel Aviv University, Tel Aviv, Israel; 13 Charite Universitätsmedizin Berlin, Berlin, Germany

**Keywords:** Sleep apnea, Central sleep apnea, Home sleep testing, Ambulatory monitoring, Peripheral arterial tone

## Abstract

**Study objectives:**

To assess the accuracy of WatchPAT (WP—Itamar-Medical, Caesarea, Israel) enhanced with a novel systolic upstroke analysis coupled with respiratory movement analysis derived from a dedicated snoring and body position (SBP) sensor, to enable automated algorithmic differentiation between central sleep apnea (CSA) and obstructive sleep apnea (OSA) compared with simultaneous in-lab sleep studies with polysomnography (PSG).

**Methods:**

Eighty-four patients with suspected sleep-disordered breathing (SDB) underwent simultaneous WP and PSG studies in 11 sleep centers. PSG scoring was blinded to the automatically analyzed WP data.

**Results:**

Overall WP apnea-hypopnea index (AHI; mean ± SD) was 25.2 ± 21.3 (range 0.2–101) versus PSG AHI 24.4 ± 21.2 (range 0–110) (*p* = 0.514), and correlation was 0.87 (*p* < 0.001). Using a threshold of AHI ≥ 15, the sensitivity and specificity of WP versus PSG for diagnosing sleep apnea were 85% and 70% respectively and agreement was 79% (kappa = 0.867). WP central AHI (AHIc) was 4.2 ± 7.7 (range 0–38) versus PSG AHIc 5.9 ± 11.8 (range 0–63) (*p* = 0.034), while correlation was 0.90 (*p* < 0.001). Using a threshold of AHI ≥ 15, the sensitivity and specificity of WP versus PSG for diagnosing CSA were 67% and 100% respectively with agreement of 95% (kappa = 0.774), and receiver operator characteristic (ROC) area under the curve of 0.866, (*p* < 0.01). Using a threshold of AHI ≥ 10 showed comparable overall sleep apnea and CSA diagnostic accuracies.

**Conclusions:**

These findings show that WP can accurately detect overall AHI and effectively differentiate between CSA and OSA.

## Introduction

Sleep apnea is an extremely common disorder, and while estimates of its prevalence depend on its definition, a recent report estimated prevalence of between 14 and 49% of middle-aged men in the USA and Europe [1] and in as many as 50% of women [2] based on a minimal apnea/hypopnea index (AHI) of five events per hour. It is further expected that this prevalence will continue to increase with the expanding global obesity epidemic. To further compound the public health implications of this alarming disease prevalence, it has been estimated that over 75% of patients with obstructive sleep apnea (OSA) are either undiagnosed or untreated [3].

The accepted gold standard for sleep apnea diagnosis is polysomnography (PSG); however, access to PSG is well-known to be limited. In view of the high prevalence of OSA, and the apparent massive shortfall in its diagnosis and treatment, and the limited availability of PSG, portable recording devices for home sleep testing (HST) have been developed for the detection of OSA [4]. Despite the considerable appeal of home testing, Yalamanchali et al. [5] observed that most such devices have not gained broad clinical appeal, due for amongst other factors to insufficient evidence supporting their accuracy. These authors performed a meta-analysis of fourteen studies using peripheral arterial tonometry (PAT)–based WatchPAT (WP) devices (Itamar Medical, Ltd.), compared with corresponding indices measured by conventional PSG [6–18]. These studies collectively provided a suitable database of 909 patients. Based upon this meta-analysis, the authors concluded that a WP technology “represents a viable alternative to PSG for confirmation of clinically suspected sleep apnea.”

One of the factors contributing to WP’s accuracy with respect to the PSG gold standard is its validated ability to accurately determine the actual total sleep time (TST) during a study using an actigraphic-based algorithm validated by simultaneous polysomnography [18–20] as opposed to the total recording time (TRC) of the study which most home testing devices use.

In addition to accurate sleep/wake discrimination, the WP has also been shown to provide accurate determinations of REM stage sleep [21, 22], as well as non-REM categorization into deep and light stages [23, 24], and further, with the addition of an optional detector located below the supra-sternal notch, provides accurate measurements of snoring and body position [25].

Although the WP has been shown to accurately identify sleep-disordered breathing (SDB) conditions, and provide a comprehensive analysis of sleep stages, so far, it has not separated between central sleep apnea (CSA) and all other types of SDB, and these events were included in its non-specific AHI.

PAT signal upstroke variations that are associated with intrathoracic pressure changes coupled with respiratory movements derived from the snoring and body position (SBP) sensor enhanced the WP algorithms to enable it to specifically detect CSA. Indeed, a recently released new version of the WP includes, in addition to its already published features, a method to specifically detect central events. We sought in the present study to validate this new capability of the WP in a multicenter study. We hypothesized that the accuracy of the WP in detecting CSA will be within the inter-scorer variability range (i.e., agreement of 0.8 and above).

## Methods

This was a multicenter study with the following centers participating:

1. Carmel Medical Center, Haifa, Israel
2. Charite Universitätsmedizin, Berlin, Germany
3. Kaiser Permanente San Jose Medical Center, USA
4. Kaiser Permanente LA Medical Center, USA
5. Kaiser Permanente Fontana Medical Center, USA
6. UF Health Sleep Center, University of Florida, USA
7. Stony Brook University Hospital, USA
8. Centre for Sleep and Chronobiology, Canada
9. Rambam Medical Center, Israel
10. Soroka Medical Center, Beer Sheva, Israel
11. Sourasky Medical Center, Tel Aviv, Israel

In all centers, an IRB approval was obtained, and all participants have signed an informed consent prior to participation. All participants underwent a full in-lab sleep study with simultaneous recording of PSG (which is in use in each center) and WP200U (Itamar-Medical, Caesarea, Israel). The WP200U signals were analyzed for a WP-derived total apnea-hypopnea index (WP AHI) and central apnea-hypopnea index (AHIc) using its automatic software and were compared with the PSG’s manual scoring which was performed centrally by an external experienced PSG technologists blinded to the WP200U analysis.

### Population selection

In order to have a substantial representation of patients with CSA within the study population, we selectively recruited heart-failure patients in this study. It was previously reported in the literature that there is a high prevalence (between 33 and 40%) of CSA in heart-failure patients [26, 27]. The total number of cardiac patients (CHF and/or AFib) included in the analysis was 50 (CHF only, *n* = 33, A. fib. only *n* = 9, and 8 patients with both conditions), comprising 59.5% of the study population. The local staff at each participating center attempted recruiting participants in whom they estimated that there was a relatively high risk of having central apneas. The contribution of the various centers was not equally distributed, and the various sites had recruited the following number of participants: 1, 2, 3, 5, 5, 8, 8, 8, 11, 12, 21 (total of 84).

The following were the inclusion and exclusion criteria in all sites:

#### Criteria for inclusion

- Age between 17 and 90
- Subject is able to read, understand, and sign the informed consent form
- Subjects suspected of having SDB with and without cardiac disorders
- Willing to sleep with the WP200U and PSG simultaneously in the sleep lab

#### Criteria for exclusion

- Finger deformity that precludes adequate sensor appliance
- Use of one of the following medications: alpha blockers, short-acting nitrates (less than 3 h before the study)

### Study population

Eighty-four (84) patients suspected of having SDB with selective bias toward recruiting patients with congestive heart failure (CHF) were recruited (54 males), age 57 ± 16 (range 22–83) years, BMI 30 ± 5.9 kg/m^2^ (range 17–45). They consented to and successfully completed the study (with at least 4 h of actual sleep time with valid signals in PSG, and at least 1.5 h of valid WP signals during actual recorded sleep).

Patient anthropometric data and medical history information are summarized in Table 1.

**Table 1:** Anthropometric characteristics and medical history parameters

	All (*n* = 84)	Male (*n* = 54)	Female (*n* = 30)
Age mean ± SD (range)	57 ± 16 (22–83)	55 ± 16 (22–83)	61 ± 16 (22–82)
Height cm mean ± SD (range)	172 ± 11.4 (150–200)	177 ± 10.0 (152–200)	163 ± 8.0 (150–182)
Weight (kg) mean ± SD (range)	89 ± 22 (49–149)	96 ± 21 (66–149)	76 ± 21 (49–129)
BMI mean ± SD (range)	29.8 ± 5.7 (17–45)	30.4 ± 5.4 (23–45)	28.6 ± 6.2 (17–39)
Hyperlipidemia *n* (%)	39 (46%)	22 (41%)	17 (57%)
Hypertension *n* (%)	51 (61%)	34 (63%)	17 (57%)
Pulmonary hypertension *n* (%)	5 (6%)	5 (9%)	0 (0%)
Diabetes *n* (%)	19 (23%)	11 (20%)	8 (27%)
Heart failure *n* (%)	41 (48%)	27 (50%)	14 (47%)
Atrial fibrillation *n* (%)	17 (21%)	10 (19%)	7 (23%)
CHF^a^ and A. fib. *n* (%)	8 (10%)	4 (8%)	4 (13%)
Pacemaker *n* (%)	4 (5%)	2 (4%)	2 (7%)

There were no significant differences in medical history by gender

^a^NYHA class II or higher

### Polysomnography

The reference used for comparison in this study was an FDA-approved in-lab PSG from multiple manufacturers used in the eleven study sites. Multichannel PSG configuration compliant with accepted standards and manual scoring according to the American Academy of Sleep Medicine (AASM) directives [28] is considered to be the “gold standard” for sleep testing, and was used to derive standard indicia including the apnea/hypopnea index (AHI) and central sleep apnea-hypopnea index (AHIc) [29, 30]. Apnea with the absence of respiratory effort was scored as central, while obstructive apnea was scored when respiratory effort was noted. In the PSG, hypopneas were scored when there was a reduction in tidal ventilation presented by a 30% or greater reduction in NAF associated with either oxygen desaturation of at least 3% or an EEG change that indicate arousal. It was classified as central or obstructive based on the presence or absence of flow limitation in the NAF signal. Obstructive hypopneas were scored when the reduction in airflow was mainly due to increased upper airway resistance (i.e., with flow limitation in the NAF signal: flattening of the inspiratory portion of the nasal pressure), while central hypopneas were scored when the reduction in flow was mainly due to a reduction in ventilatory effort (and absence of flattening of the inspiratory portion of the nasal pressure).

Enrolled patients underwent a standard sleep study using the PSG system simultaneously with the WP in the clinical sleep lab. The WP provided a series of synchronizing pulses to an auxiliary input channel of the PSG to time-synchronize-scored epochs between the two systems.

### WatchPAT system

The WP system used in this study is a home sleep testing (HST) system based on a wrist-worn device and a finger probe which acquires Peripheral Arterial Tone (PAT) signals and arterial oxygen saturation levels, together with actigraphy data from a 3D accelerometer that is embedded in the wrist unit, and an optional snoring and body position (SBP) sensor that is positioned under the sternal notch. The WP algorithm detects offline apnea/hypopnea events, respiratory effort-related arousals, and sleep/wake status, and determines sleep stages [8, 21–24].

The WP finger probe is designed to optimize the dynamic range of peripheral arterial volume changes by applying a near diastolic level of counter pressure sufficient to unload arterial wall tension, while preventing the occurrence of venous blood pooling in the finger so as to avoid the possible induction of venoarteriolar reflex–mediated vascular responses, and other measurement-related artifacts, as previously described [8, 31].

The probe measurement site at the distal phalanx of the finger is characterized by a large dynamic blood flow response range by virtue of a dense vascular network of predominantly arteriovenous shunt vessels, which are controlled by α-adrenergic receptors of vascular smooth muscle of the finger vascular bed and thus reflect sympathetic nervous system activity of the patient [10, 32, 33].

The WP assesses changes in vascular tone at the fingertip, with transient vasoconstrictory events being associated with sympathetic activation that typically terminates sleep-disordered breathing events [31]. The autonomic basis for the transient vasoconstrictory events provide a sensitive and accurate means for detecting arousals, which may in fact be considerably more sensitive than electroencephalographically detected cortical arousals. Such autonomic, brainstem, or subcortical arousals are particularly more sensitive markers of arousal even in children than EEG, as reported by Tauman et al. [34]. In adults too, it has been reported that a substantial percentage of respiratory events are not associated with cortical arousals but are related to subcortical (autonomic) arousals. This is consistent with findings in adults of absent cortical arousals in 10–25% of actual apneic events, as reported by Eckert et al. [35]. In the current study, the WP system included also the SBP placed below the sternal-notch [36].

The SBP sensor encapsulates together a microphone and a 3D accelerometer used to derive the spatial body orientation relatively to the vertical gravity from which district position can be calculated (supine, left side, right side, prone, or sitting). The microphone and a proprietary transfer function provide snoring level in decibels calibrated to an estimated recording from a 1-m distance from the patient’s head.

### Detecting central apnea with the WP

The current WP used in this study is a further development that allows the detection of central sleep apnea events. This development essentially comprised two main independent sources of physiological information that are used to discriminate between central and obstructive apneas. The first involves the use of the 3D accelerometer of the SBP sensor to derive upper chest wall respiratory movements that are diminished during a central sleep apnea event. The second is based on information derived solely from the PAT signal waveform by analyzing dynamic changes of measured systolic upstrokes as described below. These two sources of data are further supported by data from the other WP channels—oximetry, wrist movement, and snoring.

### Accelerometry-based respiratory effort sensing

The SBP sensor that contains a triaxial accelerometer was applied to the upper patient’s chest, below the supra sternal notch, and connected to the wrist-mounted WP device. Signal acquisition, signal conditioning, and signal processing yielded accelerometric data indicative of the level of respiratory effort-related chest wall motion. This information was used to determine an adaptive threshold value indicative of the presence or absence of respiratory effort during apneic events determined by the WP system, and thus allows for the identification of central events. The detection was done by a multidimensional classifier, which involved a cumulative indication from several signals, and a few characteristics that disqualified an event from being central.

**Fig. 1:**
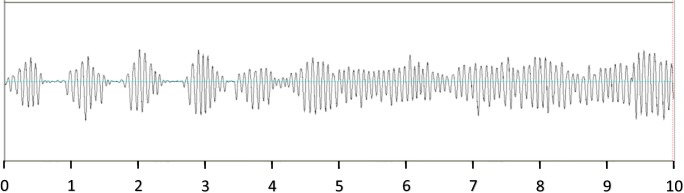
Chest wall movement recorded by RESBP. A 10-min recording of the WP respiratory movement sensor, illustrating recurrent periods of absent respiratory effort during central apneic events, interspersed by increased effort, followed by continuous respiratory movement during an apnea-free period

A representative 10-min example of the output signal of this sensor is shown in Fig. 1. This clearly shows the recurrent absence of respiratory effort during central apneic events, followed by continuous respiratory movement during an apnea-free period.

The use of an accelerometer for determining the presence, stability, and magnitude of chest wall motion indicative of respiratory effort can provide a robust method for differentiation of central and obstructive apneas, as has been previously reported [37–39].

### Finger sensor–based pulse upstroke detection

The WP central apnea detection method which is based on plethysmographic information derived from the finger sensor provides a method for determining the effect of respiratory effort on the PAT signal’s pulse wave.

This is based on an interaction between breathing-related changes of intrathoracic pressure and the dynamic changes occurring during ventricular systole on the detected pulse-wave signals, which occur for amongst other reasons, because the thoracic pressure changes directly impinge on the heart.

The method entails (a) the detection of successive systolic upstrokes; (b) the determination of an index of upstroke dynamics, for example, the fraction of the upstroke occurring within a prescribed time interval commencing at a prescribed fraction of the upstroke or the slope between preset fractions of the upstroke; (c) the normalization to changes within a certain time window; and (d) the determination of the variability of a series of upstroke dynamic index values in time corresponding to the patients breathing cycle.

Essentially, this enables a clear distinction to be made between central and non-central apneas as the two respective conditions represent polar opposites in the degree of breathing-related thoracic pressure–induced changes on the pattern of the pulse-wave upstroke. In the case of central apnea, the absence of respiratory effort means that the stereotypical pattern of the systolic upstroke is unaffected by superimposed pressure fluctuations due to respiratory effort.

In contrast, the large intrathoracic pressure swings associated with resistive breathing during upper airway obstruction or partial obstruction result in changes in the pattern of the systolic upstroke, which vary throughout the course of each breathing cycle, and result in a characteristic, cyclic pattern of changes in the upstroke dynamics over time.

**Fig. 2:**
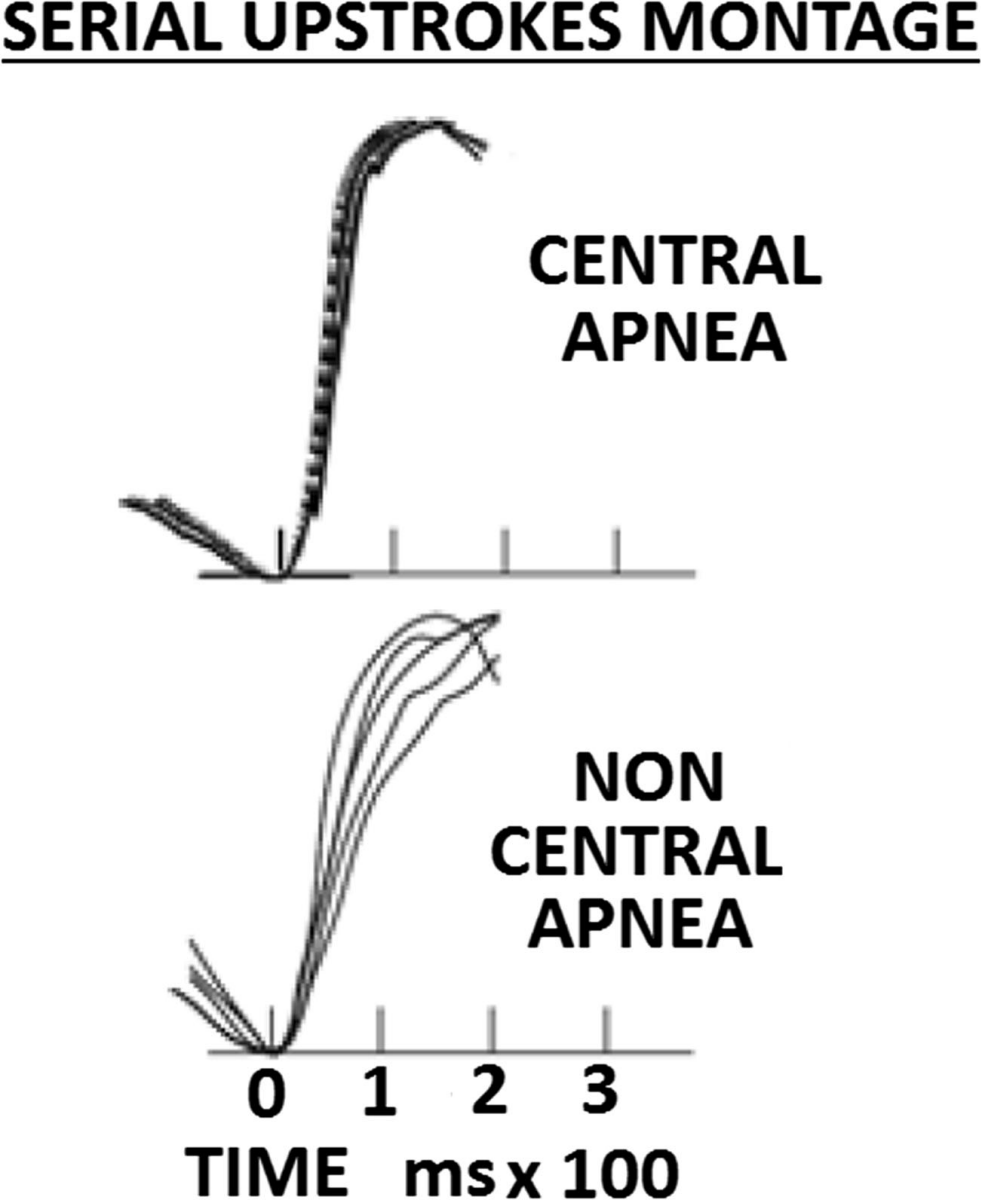
Comparative montages of systolic upstrokes in central and non-central apnea. Upper trace—a montage of superimposed pulses during a central apnea illustrating a low level of variability of the normalized systolic upstroke waveforms. Lower trace—the contrasting pattern of systolic upstrokes during non-central apneas illustrating a high level of variability. In both cases, pulses are synchronized at the foot of the upstroke signals

The contrasting patterns of PAT upstrokes during central and non-central apneas can be seen in a montage of superimposed pulses in Fig. 2.

**Fig. 3:**
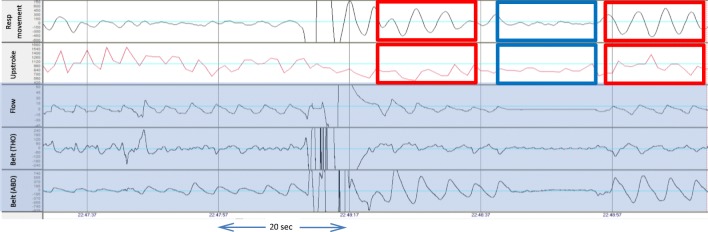
Time course of PSG signals and WP respiratory movement and systolic upstroke index signals. Time-course of respiratory signals during central apnea events in a patient. Signals, from top to bottom, are respiratory chest movement, upstroke index, nasal air flow, and abdominal belt (ABD). Red boxes indicate normal breathing while the blue boxes indicate the reduced respiratory chest wall movement and upstroke index during a central apnea

Figure 3 shows the time course during central apnea events in a patient. The signals shown from top to bottom are the respiratory chest movement and upstroke index, which are derived from the WatchPAT device, as well as flow and thoracic and abdominal belts taken from the PSG (marked with darker background).

As can be seen, the respiratory movement is similar to the belt and shows reduction in the movement during apneic events only. Both respiratory chest movement signal and PAT upstroke index pattern variability are substantially reduced during the central event (marked by blue box). The red boxes mark normal breathing before and after the event.

### Combined accelerometry and upstroke analysis and WatchPAT algorithm-algorithm tuning

Both the PAT signal upstroke variations associated with intrathoracic pressure changes, and the respiratory movements derived from the enhanced SBP sensor are used to identify central apnea/hypopnea events in a newly upgraded algorithms of the WP software. In addition to these two inputs, additional WP information is used in the algorithm to support the detection: (a) the absence of snoring and (b) decrease in breathing movement as reflected in hand movement that is measured in the wrist actigraphy, if such movement was present before and after the event. Prior to the present study, the algorithm parameters utilizing these parameters were tuned in an optimizing process in a training set of 69 cardiac and OSA patients and were shown to give robust correlation with polysomnography CSA detection results. This algorithm was used in the present study.

### The hierarchy of CSA detection

The WP determination of SDB events is performed in hierarchical order:

- Step 1—detection of all SDB events and calculation of AHI.
- Step 2—calculation of upstroke variability and chest movement signal.
- Step 3—applying the above to identify the central events out of all events.

### Data analysis and statistical methods

#### Primary analyses

Two parameters obtained from both the WP200U device and the gold standard PSG were compared: AHI and AHIc. All parameters were compared as continuous variables and as dichotomous variables, provided from respective thresholds of 10 and 15, for sensitivity analysis. Subjects’ demographic characteristics were presented as mean ± SD and comparison by gender were demonstrated using an independent *t* test. Subjects’ medical history parameters were presented as frequency and percentage, and comparison by gender was demonstrated using chi-square test. Comparisons between parameters obtained from the WP200U device and from PSG were made using paired *t* tests. For sensitivity, specificity, PPV, and NPV, the WP200U parameters were compared with the PSG gold standard based on thresholds of 10 and 15. In addition, areas under the curve (AUC) of ROC curves with PSG thresholds of 10 and 15 were calculated. Agreement of parameters measured by the WP200U versus PSG was computed using overall agreement and kappa level of agreement. For correlation analysis, a Pearson correlation was used and scatter graphs and linear regressions coefficients were generated.

## Results

The results are based on data obtained from a validation set of 84 subjects, 54 males and 30 females. Since we found no gender-specific changes in the WatchPAT detection of SDB, genders are combined and presented as a whole. The mean age was 57 ± 16 (range 22–83) with no differences in age and BMI between genders (Table 1). Patient’s medical history presented high percentages of hyperlipidemia, hypertension, heart failure, and diabetes. There were no significance differences in comorbidities between genders (Table 1).

Table 2 provides summary statistics for overall total of the AHI, AHIc, and sleep times for the WP and PSG, respectively, as well as within-patient statistics of significance of differences, and of the respective correlations. No significant differences in the AHIc were found between the 17 patients designated as having A. fib. and the 67 non-A. fib. patients, in both the PSG and WatchPAT, nor were there any significant differences between corresponding matched PSG versus WatchPAT AHIc in the respective A. fib.–positive and A. fib.–negative groups (in contrast to the overall lumped population (*p* < 0.05). When the population was partitioned according to a BMI above or below 30 kg/m^2^, significantly higher values of AHIc and obstructive AHI values were found in the BMI ≥ 30 group, in both the PSG and WatchPAT analyses. Within each BMI group, there were no significant differences between the WatchPAT and PSG indices of AHIc or obstructive AHI.

**Table 2:** Summary statistics comparing WP and PSG measurements, sleep time, and SDB indices

	WP (*n* = 84)	PSG (*n* = 84)	WP versus PSG (*p*)	Correlation *R* (*p*)
Sleep time (h)	5.658 ± 1.10	5.645 ± 1.05	0.897	0.608 (*p* < 0.00001)
AHI mean ± SD (range)	25.2 ± 21.3 (0.2–101)	24.4 ± 21.2 (0–110)	0.514	0.873 (*p* < 0.00001)
AHIc mean ± SD (range)	4.2 ± 7.7 (0–38)	5.9 ± 11.8 (0–63)	0.034	0.799 (*p* < 0.00001)

Sleep time and overall AHI did not demonstrate significant statistical differences between the WP and PSG measurements, while the central AHI parameter differed significantly between methods. AHI and AHIc, as well as sleep time, were all highly correlated between the WatchPAT and PSG.

**Fig. 4:**
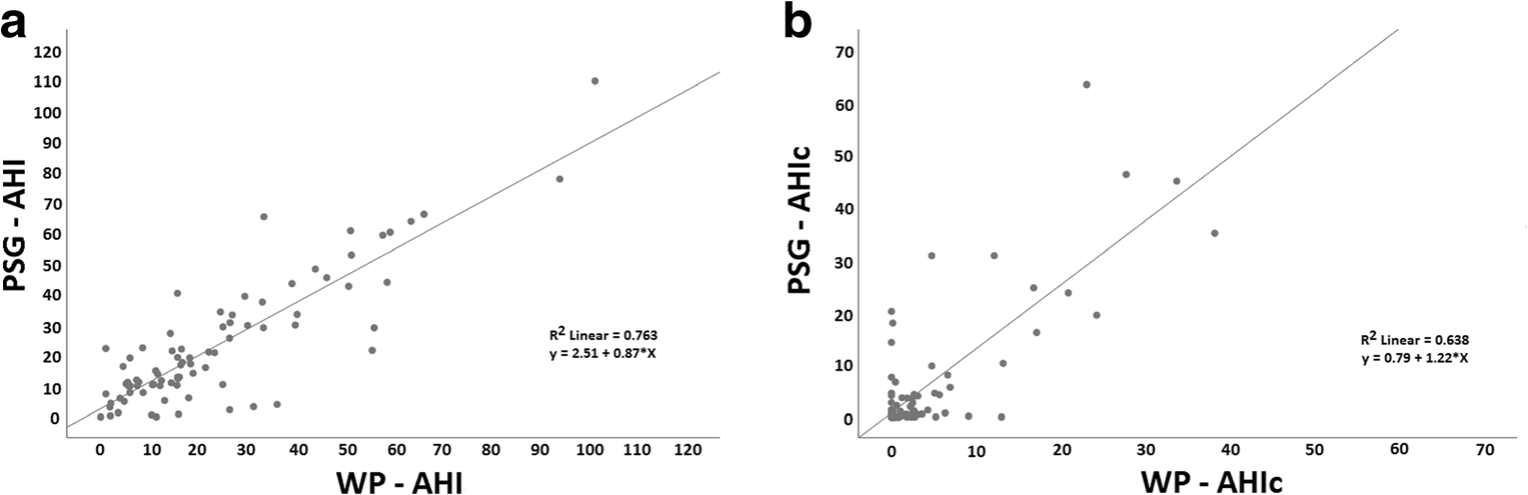
Scatterplot produced by WatchPAT and PSG of total AHI (**a**) and central AHIc (**b**). Scatterplots of PSG (*y*-axis) versus WP (*x*-axis), illustrating the correlations between **a**—overall apnea/hypopnea index values (*r* = 0.87, *P* < 0.001) and **b—**central apnea/hypopnea index values (*r* = 0.80, *P* < 0.001)

The highly significant correlations between sleep-disordered conditions can be appreciated in the scatter plots shown in Fig. 4a, b, which plot sleep apnea measurements produced by the WatchPAT and PSG, respectively, and demonstrate the clear correlations between total AHI (Fig. 4a) and central AHI (Fig. 4b).

Applying the Kolmogorov-Smirnov test for normality, we observed a skewed distribution for all parameters (*P* < 0.001 for all). Therefore, we used Spearman’s correlation to demonstrate the relationship between the WatchPAT and PSG measurements. There were high and significant relationship between the two measurements in all sleep apnea parameters (*P* < 0.00001 for all). All slopes were not significantly differing from 1 (Fig. 4).

**Fig. 5:**
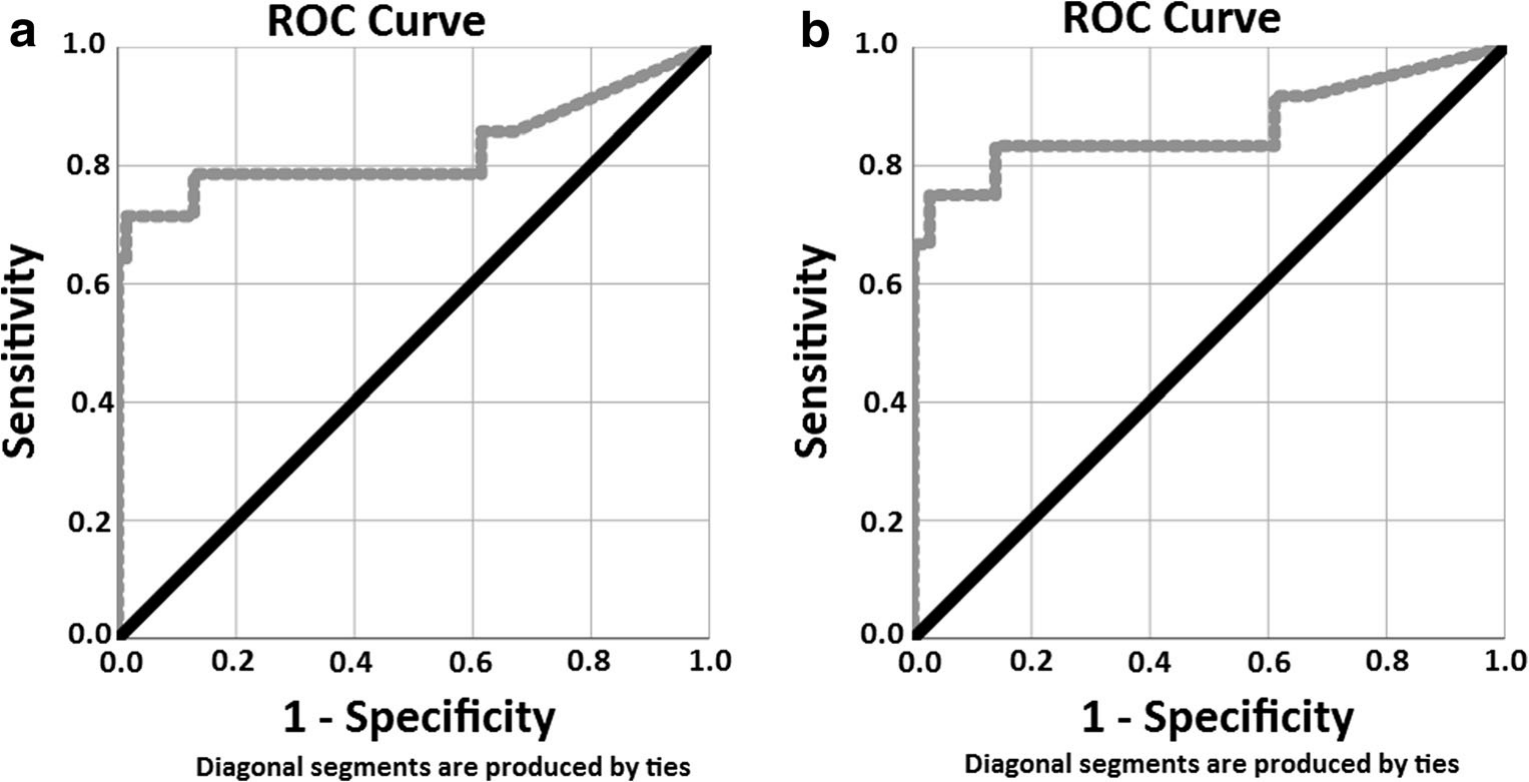
ROC curves WP versus PSG when utilizing thresholds of 10 (**a**-left) and 15 (**b**-right) for AHIc. Comparative receiver operating curves (ROC) of WP central events for thresholds of 10 (left) and 15 (right), of the PSG gold standard. **a** AUC = 0.827, kappa = 0.557; *P* < 0.001. **b** AUC = 0.866, kappa = 0.384; *P* < 0.001

To further assess the clinical utility of the WatchPAT-based detection of central sleep apnea events, the ROC curves for PAT device measurements when utilizing clinically relevant thresholds of 10 and 15 events per hour for PSG measurements are shown in Fig. 5a (threshold 10/h) and Fig. 5b (threshold 15/h). The areas under the curve (AUC) for AHIc was significantly high in both cases (AUC = 0.827 and 0.866, respectively).

Table 3 summarizes the sensitivity, specificity, PPV and NPV, agreement, kappa, and area under the ROC curve data for thresholds of 10 and 15 events/h, respectively, and provides data for total AHI and central AHI measurements. The sensitivity and PPV for AHI and AHIc were significantly high in both cases.

**Table 3:** Agreement between PAT and PSG measurements (cut points = 10/h and 15/h)

SDB/threshold	Sensitivity (%)	Specificity (%)	PPV (%)	NPV (%)	Area under ROC curve (*p*)
Total AHI ≥ 10 events/h	83.3	55.6	87.3	47.6	0.791 *P* < 0.001
Total AHI ≥ 15 events/h	85.1	70.3	78.4	78.8	0.867 *P* < 0.001
AHIc ≥ 10 events/h	71.4	98.6	90.9	94.5	0.827 *P* < 0.001
AHIc ≥ 15 events/h	66.7	100	100	94.7	0.866 *P* < 0.001

**Fig. 6:**
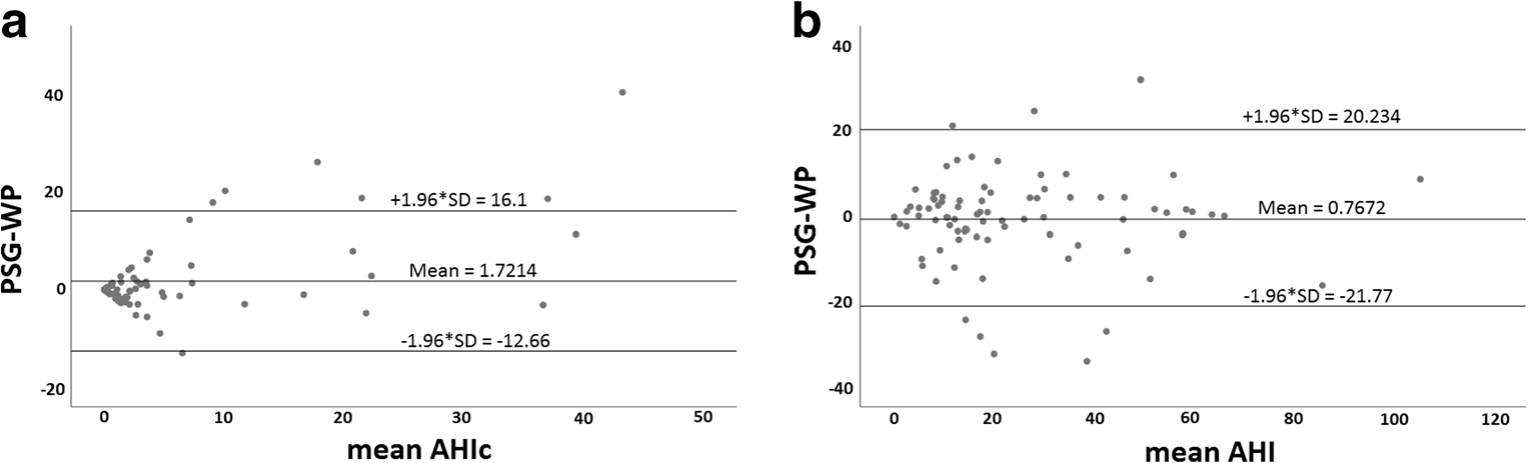
**a** Bland-Altman plot for the effect of mean AHI on the difference in AHI between WatchPAT and PSG. **b** Bland-Altman plot for the effect of mean AHIc on the difference in AHI between WatchPAT and PSG. Bland-Altman plots for the effect of mean AHI (**a**) or mean central AHI, (**b**) on the differences between WatchPAT and PSG. As can be seen, on the average across various severities of AHI, PSG scored 0.77 less events per hour of sleep compared with WP, while for AHIc PSG scored 1.7 events greater than WP per hour of sleep. In both cases, there was no substantial effect of the average AHI severity on the difference between the methods

To assess the agreement between the methods, we used the Bland-Altman analysis and evaluated the bias between the mean differences and estimated the agreement interval, within which 95% of the differences fall. For total AHI, mean difference between methods was − 0.77 ± 10.7 (SD), and for central AHIc, mean difference between methods was 1.72 ± 7.3 (SD) (Fig. 6).

## Discussion

### Overview of findings

Based on simultaneous comparisons to the PSG gold standard in 84 patients in multiple centers, our findings support our hypothesis that the WatchPAT, when enhanced with the two new features, PAT signal upstroke analysis and RESBP, can accurately detect overall AHI and AHIc, all with correlations of 0.80 or higher compared with in-lab PSG.

In addition, the WP further benefits from the well-established advantages of home sleep testing, including the realistic option of tracking patient condition over time as well as in response to therapeutic interventions. In particular, the ability to accurately diagnose CSA using a very simple and patient-friendly system may offer considerable advantages for a patient population when considering treatment.

### Accelerometry-based respiratory effort sensor

Although accelerometric-based detection of chest wall movement is a well-known method for sensing respiratory effort [37–39], it has not been widely integrated into clinical use, and respiratory belts are the common standard. Although placement of the sensor makes patient setup somewhat complicated, it still provides a more robust and comfortable attachment than respiratory belts which tend to slide and become displaced and may therefore be advantageous for home sleep testing. The use of SBP in which the sensing element (accelerometer) is already in place obviates the need for a dedicated effort device, whether a belt or another accelerometric sensor and as such does not add any additional complexity. A potential disadvantage of the SBP in comparison with the other methods is its location on the very upper chest where respiratory movement is subtle and therefore the signal-to-noise ratio is lower.

### Physiological basis of pulse upstroke algorithm

In a somewhat similar approach to using the peripheral pulse wave to differentiate between central and non-central events during sleep to that used in this study, Sommermeyer et al. [40] also used a peripheral pulse signal to determine the degree of respiratory effort during apneic events in order to determine the nature of the events, and to differentiate central from non-central events.

These authors determined the signal power in the typical frequency band of breathing (0.15–0.4 Hz) during the apnea phase and compared it with the signal power of the same frequency range 15 s before the apnea event to thereby define an effort ratio index.

In the present study, we restricted the pulse analysis to the duration of the systolic upstroke and normalized the amplitude of the pulse waves in order to avoid the potentially confounding influence of spontaneous modulation of the pulse amplitude, which may occur at variable frequencies and with large and variable amplitude changes, as observed by Burton [41].

In addition, variations in the finger pulse amplitude frequency domain occur during sleep and have in fact been used by the WP to discriminate between REM and non-REM sleep [22] and between light and deep non-REM sleep stages [23, 24].

### Selection of cut point and rational for the chosen values

We used 2 AHIc thresholds to demonstrate the accuracy of WP’s diagnosis, 10 events/h and 15 events/h. Gabor et al. [42] has chosen 10 events/h cut-off point stating that “Although no formal determination regarding the definitive threshold has been set, the AHI ≥ 10/h is commonly used to establish a sleep apnea diagnosis, and AHIc > 10 to differentiate the diagnosis as central,” and same approach was suggested by Cowie et al. [43, 44] in the SERVE-HF study who also used this threshold to enroll predominantly CSA participants defined as having AHI ≥ 15 events per h, with > 50% central events and a central AHI of ≥ 10 events per h. However, an AHIc of 15-events/h threshold is more commonly used in the clinical setting and serves to approve treatment, and may represent a clinical threshold of amenability to improvement following treatment [45–47]. Furthermore, Arzt et al. [46] reported that “early suppression of CSA by CPAP to an AHI below 15 per h may improve both LVEF and transplant-free survival.”, and likewise, Momomura [47] found that in CSA patients in whom AHI was improved to below 15, CPAP treatment resulted in a greater increase in LV ejection fraction at 3 months and significantly better transplant-free survival than control subjects, whereas in those whose AHI remained above 15, neither LV ejection fraction or survival was improved.

### Limitations

The two approaches for determining central apneas used in this study have limitations. Finger vascular responses may be adversely affected by the presence of cardiac arrhythmia, which can affect the effectiveness of myocardial contraction and thus introduce upstroke index variability unrelated to the changes in intrathoracic pressure associated with respiratory effort. Such changes would however be easily recognized on the basis of the inter-beat timing, and in the case of reproducible arrhythmias, the effect on upstroke dynamics could be possibly be learned, and adjusted for.

In the case of the accelerometric evaluation of chest wall motion indicative of respiratory effort, similarly to other surface-attached technologies in use, it is possible that chest wall motion unrelated to breathing or respiratory effort may confound the analysis. Similarly, variations in patient posture may alter the basic conditions for signal transduction, and change the signal gain and/or frequency characteristics. The accuracy of sensor placement and coupling to the body surface may also be susceptible to the limitations imposed by patient self-application.

The combined use of both independent methods in this study may have reduced the potential inaccuracies of each respective method.

The selection of a patient population with high percentage of heart failure in this study may have resulted in an unrepresentative sample of the central sleep apnea patient population at large. However, irrespective of the etiology of CSA, the functional outcome of absent respiratory effort remains a common feature, and the diagnostic principles employed in this study were explicitly directed to identifying absent respiratory effort.

## Conclusion

The present study evaluated the ability of the WatchPAT, a simple-to-apply and minimally disruptive ambulatory sleep testing device, to accurately detect central sleep apnea and total sleep apnea events during sleep.

Although central sleep-disordered conditions are much less common than their obstructive counterparts, accurate discrimination between these conditions is important, since central events are associated with increased morbidity and mortality in heart-failure patients and may have important clinical implications. Furthermore, differentiating the nature of sleep-disordered breathing is very important in determining the kind of treatment which is appropriate to the condition.

The present validation study has shown that accurate detection of central apnea, without compromising the accuracy of the general SDB assessment, is indeed possible using the WatchPAT, making this a realistic objective for home sleep testing. Future studies to include larger and diverse populations of patients reflecting a broad spectrum of central apnea severity should be undertaken to confirm these findings.

## List of abbreviations

AHI: Apnea-hypopnea index
AHIc: Central apnea-hypopnea index
AUC: Area under the receiver operating curve
CSA: Central sleep apnea
HST: Home sleep testing
OSA: Obstructive sleep apnea
PSG: Polysomnography
RES: Respiratory movements
RESBP: Respiratory movements and snoring and body position
ROC: Receiver operating curve
SDB: Sleep-disordered breathing
SBP: Snoring and body position
WP: WatchPAT
WP AHI: WP apnea-hypopnea index
